# Evolution of Costs of Inflammatory Bowel Disease over Two Years of Follow-Up

**DOI:** 10.1371/journal.pone.0142481

**Published:** 2016-04-21

**Authors:** Mirthe E. van der Valk, Marie-Josée J. Mangen, Mirjam Severs, Mike van der Have, Gerard Dijkstra, Ad A. van Bodegraven, Herma H. Fidder, Dirk J. de Jong, C. Janneke van der Woude, Mariëlle J. L. Romberg-Camps, Cees H. M. Clemens, Jeroen M. Jansen, Paul C. van de Meeberg, Nofel Mahmmod, Andrea E. van der Meulen-de Jong, Cyriel Y. Ponsioen, Clemens Bolwerk, J. Reinoud Vermeijden, Peter D. Siersema, Max Leenders, Bas Oldenburg, COIN study group and the Dutch Initiative on Crohn and Colitis

**Affiliations:** 1 Department of Gastroenterology and Hepatology, University Medical Centre Utrecht, Utrecht, the Netherlands; 2 Julius Centre for Health Sciences and Primary Care, University Medical Centre Utrecht, Utrecht, the Netherlands; 3 Department of Gastroenterology and Hepatology, University Medical Centre Groningen, Groningen, the Netherlands; 4 Department of Gastroenterology and Hepatology, VU University Medical Centre, Amsterdam, the Netherlands; 5 Department of Internal Medicine. Gastroenterology and Geriatrics, Atrium-Orbis Medical Centre, Heerlen-Sittard-Geleen, the Netherlands; 6 Department of Gastroenterology and Hepatology, Radboud University Medical Centre Nijmegen, Nijmegen, the Netherlands; 7 Department of Gastroenterology and Hepatology, Erasmus University Medical Centre, Rotterdam, the Netherlands; 8 Department of Gastroenterology and Hepatology, Diaconessenhuis, Leiden, the Netherlands; 9 Department of Gastroenterology and Hepatology, Onze Lieve Vrouwe Gasthuis, Amsterdam, the Netherlands; 10 Department of Gastroenterology and Hepatology, Slingeland Hospital, Doetinchem, the Netherlands; 11 Department of Gastroenterology and Hepatology, Antonius Hospital, Nieuwegein, the Netherlands; 12 Department of Gastroenterology and Hepatology, Leiden University Medical Centre, Leiden, the Netherlands; 13 Department of Gastroenterology and Hepatology, Academic Medical Centre Amsterdam, Amsterdam, the Netherlands; 14 Department of Gastroenterology and Hepatology, Reinier de Graaf Groep, Delft, the Netherlands; 15 Department of Gastroenterology and Hepatology, Meander Medical Centre, Amersfoort, the Netherlands; CWRU/UH Digestive Health Institute, UNITED STATES

## Abstract

**Background:**

With the increasing use of anti-TNF therapy in inflammatory bowel disease (IBD), a shift of costs has been observed with medication costs replacing hospitalization and surgery as major cost driver. We aimed to explore the evolution of IBD-related costs over two years of follow-up.

**Methods and Findings:**

In total 1,307 Crohn's disease (CD) patients and 915 ulcerative colitis (UC) patients were prospectively followed for two years by three-monthly web-based questionnaires. Changes of healthcare costs, productivity costs and out-of-pocket costs over time were assessed using mixed model analysis. Multivariable logistic regression analysis was used to identify costs drivers. In total 737 CD patients and 566 UC were included. Total costs were stable over two years of follow-up, with annual total costs of €7,835 in CD and €3,600 in UC. However, within healthcare costs, the proportion of anti-TNF therapy-related costs increased from 64% to 72% in CD (p<0.01) and from 31% to 39% in UC (p < 0.01). In contrast, the proportion of hospitalization costs decreased from 19% to 13% in CD (p<0.01), and 22% to 15% in UC (p < 0.01). Penetrating disease course predicted an increase of healthcare costs (adjusted odds ratio (adj. OR) 1.95 (95% CI 1.02–3.37) in CD and age <40 years in UC (adj. OR 4.72 (95% CI 1.61–13.86)).

**Conclusions:**

BD-related costs remained stable over two years. However, the proportion of anti-TNF-related healthcare costs increased, while hospitalization costs decreased. Factors associated with increased costs were penetrating disease course in CD and age <40 in UC.

## Introduction

Crohn’s disease (CD) and ulcerative colitis (UC), collectively known as inflammatory bowel diseases (IBD), are characterized by chronic relapsing intestinal inflammation that may lead to severe complications and disability. Therefore, IBD represent a high economic burden to society.[1–8] The early onset and chronicity of IBD profoundly affects work productivity with accompanying economic losses mainly resulting from sick leave and work disability accounting for up to 50% of the total costs.[1;2;5–8]

With the introduction and increasing use of anti-TNF therapy in IBD, a major shift of costs has been observed with medication costs replacing in-patient care, such as hospitalization and surgery, as the greatest source of healthcare expenditure.[1] Most previous cost studies in IBD, however, relied on a single measurement of costs and were performed before the introduction of anti-TNF therapy in IBD.[2;3;7–10] Furthermore, only a limited number of studies have aimed to identify factors predicting IBD-related costs.[1;4;10;11]

The ‘Costs Of Inflammatory bowel disease in the Netherlands’ or COIN-study has been initiated to generate longitudinal cost data in order to assess the impact of anti-TNF therapy on IBD-related costs. In the present study we aimed 1) to assess the evolution of costs of IBD over a period of two years, 2) to explore the contribution of healthcare, productivity and out-of-pocket costs on IBD-related costs; and 3) to identify predictors for high costs over two years of follow-up.

## Material and Methods

### Study design and patient population

**Fig 1:**
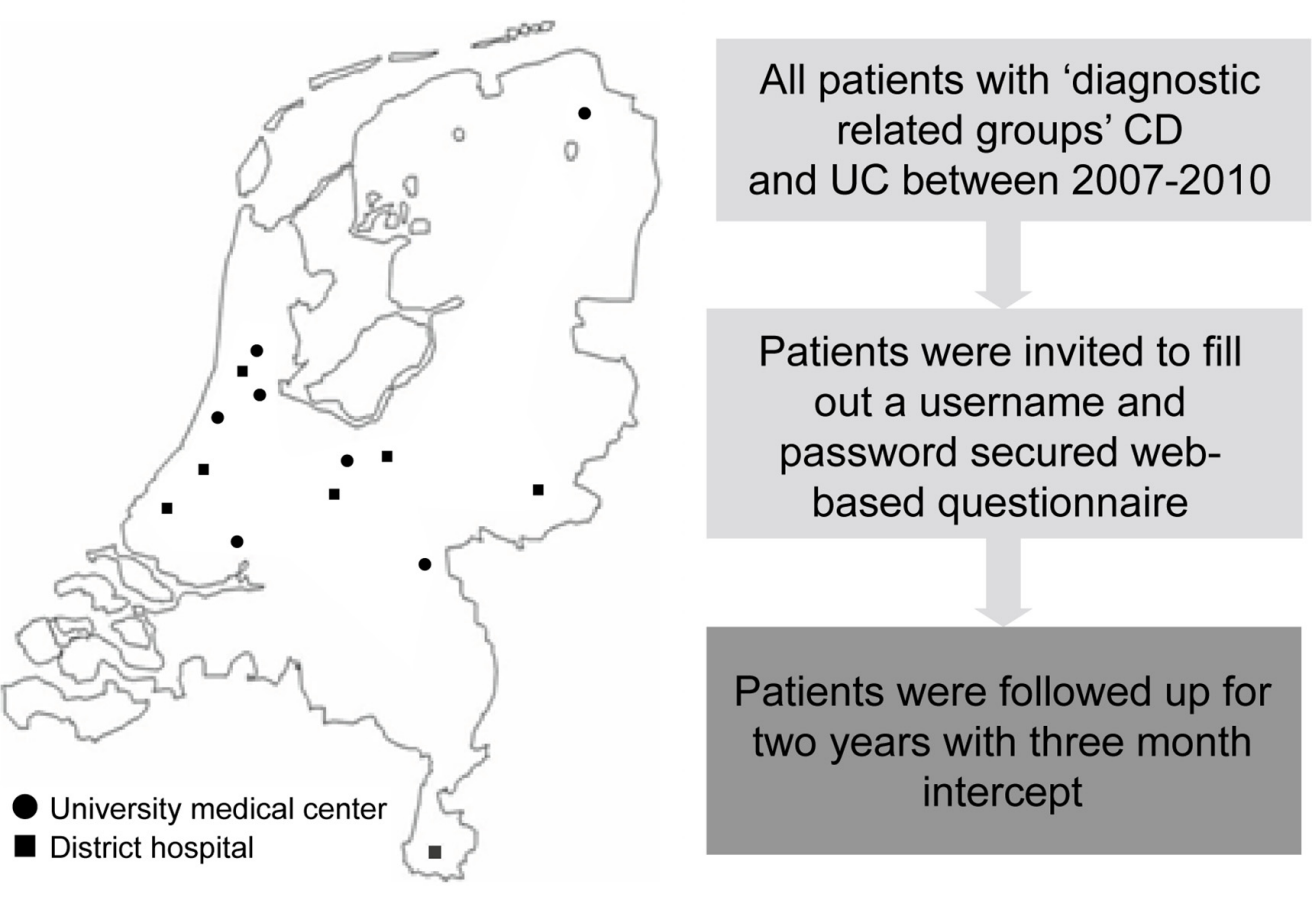
Design of the COIN study.

From October 2010 to October 2011 we invited all IBD patients aged 18 years or older from seven university hospitals and seven district hospitals to participate in the COIN-study by letter (Fig 1).

A secure web-based questionnaire was developed to obtain baseline characteristics and collect cost data on a three-month basis during two years of follow-up. The cohort organisation and study follow-up protocol have been described in detail elsewhere.[1] The study was centrally approved by the Ethics Committee of the University Medical Centre Utrecht.

### Data collection

Demographic characteristics included gender, age, age at diagnosis, education level, work status, family history, and smoking status. Clinical characteristics included subtype of IBD, disease duration and localization, disease behaviour, stoma or pouch surgery, and clinical disease activity.

In accordance with Drummond et al.[12], we distinguished three main IBD-related cost categories including healthcare costs, productivity losses and patient costs. Applying the human capital approach, productivity losses were estimated by multiplying the self-reported number of sick leave days from both paid and unpaid (i.e. voluntary work) work of patients and the caregivers taking care of the sick persons by age- and sex-specific productivity losses. A work-week was assumed to have at maximum of five working days. Patient costs were calculated according to patient specifications. Reference prices used in the COIN-study are described in S1 Table. All costs are expressed in 2011 euros, using Dutch consumer price index when appropriate. No discounting was applied, given the limited follow-up period of two years. Potential predictive variables were identified from earlier studies on predictors for poor clinical outcome or high healthcare-or productivity losses (S2 Table).

### Statistical analysis

Data analysis was performed using SPSS version 18.0. Descriptive statistics were used to characterize patients with CD and UC. We report means with a standard deviation (SD) and medians with an interquartile range (IQR). Comparisons between CD and UC patients were analysed with Student’s t-test for continuous variables and χ2 for dichotomous variables. To compare medians, the Mann-Whitney U test was used. Costs were reported as mean cost/patient with a 95% confidence interval.

To control equality between the study population (i.e. responders) and the patients who were lost to follow-up over time (i.e. non-responders) we performed a non-responder study. To account for missing data and repeated measurements, we used a generalized mixed model to compare costs between different subgroups.

We performed a multivariate logistic regression analysis to identify factors predicting increase of healthcare costs over two-years of follow-up. As a dependent variable we used the 10 percent of patients who displayed the highest increase in healthcare costs over two years of follow-up. Variables that reached borderline significance (p<0.1) in the univariate analysis were considered for inclusion into the multivariate models. We fitted separate models for UC and CD. P-values <0.05 were considered statistically significant.

## Results

### Study population

At baseline, 1,307 CD patients and 915 UC patients were included. The two-year follow-up questionnaire was filled-out by 736 CD patients and 566 UC patients (response rates of 47% and 54%, respectively). Additional response rates per time point are provided in S3 Table. From the patients who were lost to follow-up, 10 subjects died during the follow-up period, 54 were unreachable due to automatic email response bouncing our request (possibly due to a change of email address), 153 withdrew consent and 1,049 were lost for unknown reasons. Responders were older (p<0.01) and had longer disease duration (p<0.01) as compared to non-responders (S4 Table).

The baseline characteristics of the study population completing the two-year follow-up are described in Table 1. CD patients were more often females (60% versus 46%, p<0.01), smokers (19% versus 8%, p<0.01), and had a higher probability of previous abdominal surgery (56% versus 19%) compared to UC patients. CD patients were more frequently treated with immunomodulators (36% versus 23%, p<0.01) and/or anti-TNF (21% versus 4%, p<0.01) as compared to UC patients.

**Table 1:** Demographic and disease characteristics of the study population. SD: Standard deviation; IQR: Interquartile range; n/a: not applicable; NS: not significant.

	CD n = 737	UC n = 566	P-value
Male gender (%)	295 (4.0)	300 (53.0)	<0.01
Age—years (± SD)	50.5 (13.5)	52.4 (12.9)	0.01
Smoking (%)			<0.01
Current	137 (18.6)	45 (8.0)	
Never	382 (51.8)	336 (59.4)	
Ex-smoker	218 (29.6)	185 (32.7)	
Low education (%)	445 (60.4)	314 (55.5)	0.08
Disease duration—median (IQR)	18.2 (10.1–18.2)	16.0 (9.0–16.0)	<0.01
Disease localisation (%)			
Large bowel	204 (27.7)	566 (100)	n/a
Small bowel	152 (20.6)		
Both small and large bowel	361 (49.0)		
Unknown	20 (2.7)		
Penetrating disease course (%)	400 (54.3)		n/a
Clinical remission (%)	618 (83.9)	452 (79.9)	0.06
Abdominal surgery (%)	416 (56.4)	106 (18.7)	<0.01
Medication use (%)			
Mesalazine	175 (23.7)	373 (65.9)	<0.01
Azathioprine	189 (25.6)	91 (16.1)	<0.01
Mercaptopurine	51 (6.9)	36 (6.4)	NS
Methotrexate	25 (3.4)	1 (0.2)	NS
Prednisone	37 (4.9)	31 (5.5)	NS
Budesonide	44 (6.0)	19 (3.4)	NS
Infliximab	72 (9.8)	14 (2.5)	<0.01
Adalimumab	85 (11.5)	5 (0.9)	<0.01

### IBD-related costs

**Fig 2:**
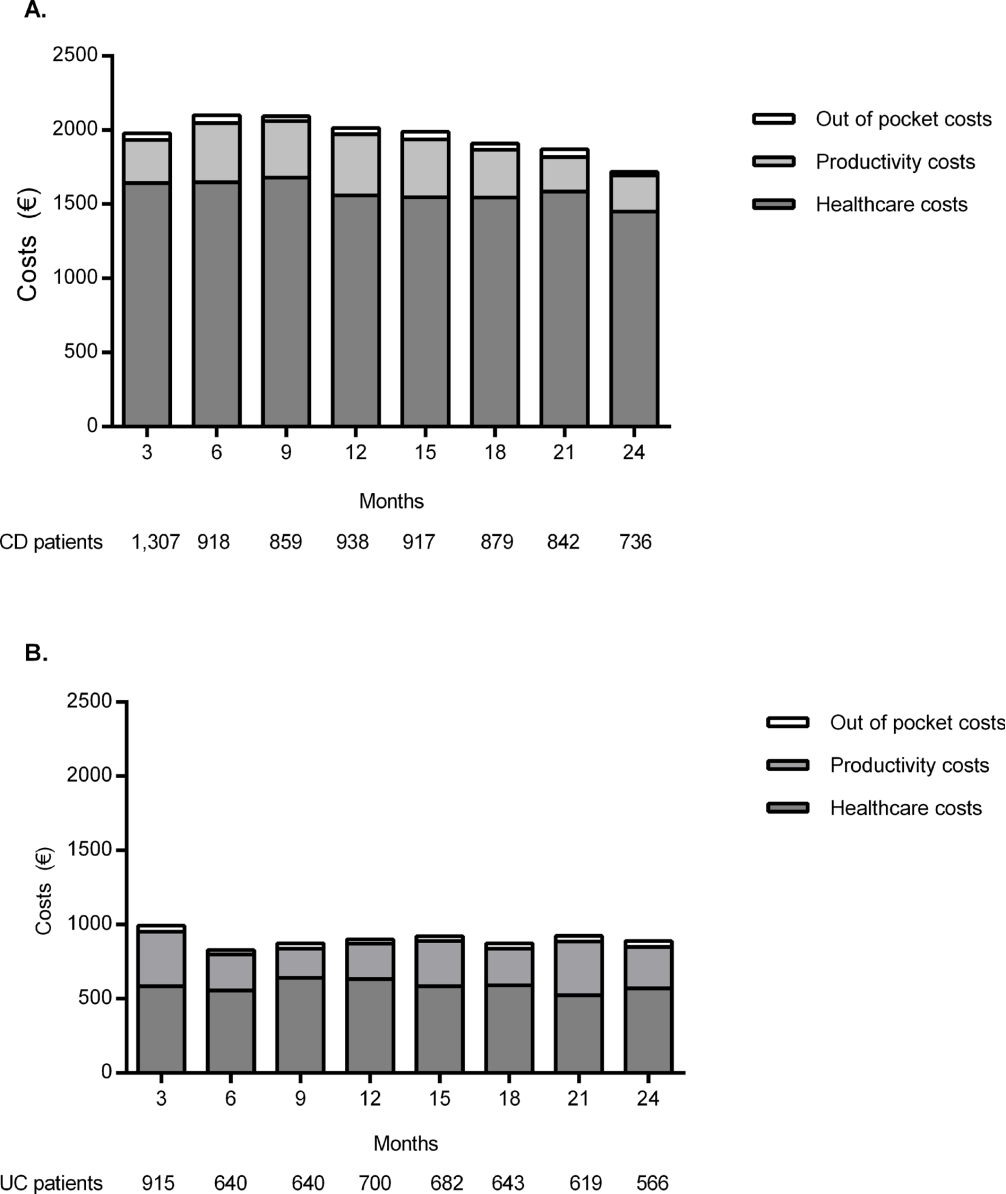
**A.** Three-monthly total costs per average CD-patient over two-year follow up. **B.** Three-monthly total costs per average UC-patient over two-year follow up.

Over the two-year follow-up period, IBD-related costs did not change (Fig 2A and 2B). The mean annual IBD-related costs were €7,835 (95% CI €7,235- €9,563) for CD patients and €3,600 (95% CI €2,865- €4,669) for UC patients. Healthcare costs accounted for the major part of the IBD-related costs, 81% (€6,326 (95% CI €5,241- €7,102)) in CD and 65% (€2,340 (95% CI €1,540- €3,105)) in UC. In addition, productivity losses accounted for 17% (€1,335 (95% CI €860- €2,130)) of the total costs in CD patients and 31% (€1,120 (95% CI €571- €1,891)) in UC patients, whereas out-of-pocket costs accounted for 2% (€174 (95% CI €95- €220) in CD and 4% (€140 (95%CI €110-€195) in UC. Associated healthcare costs per 3 months are displayed in S5A and S5B Table.

**Fig 3:**
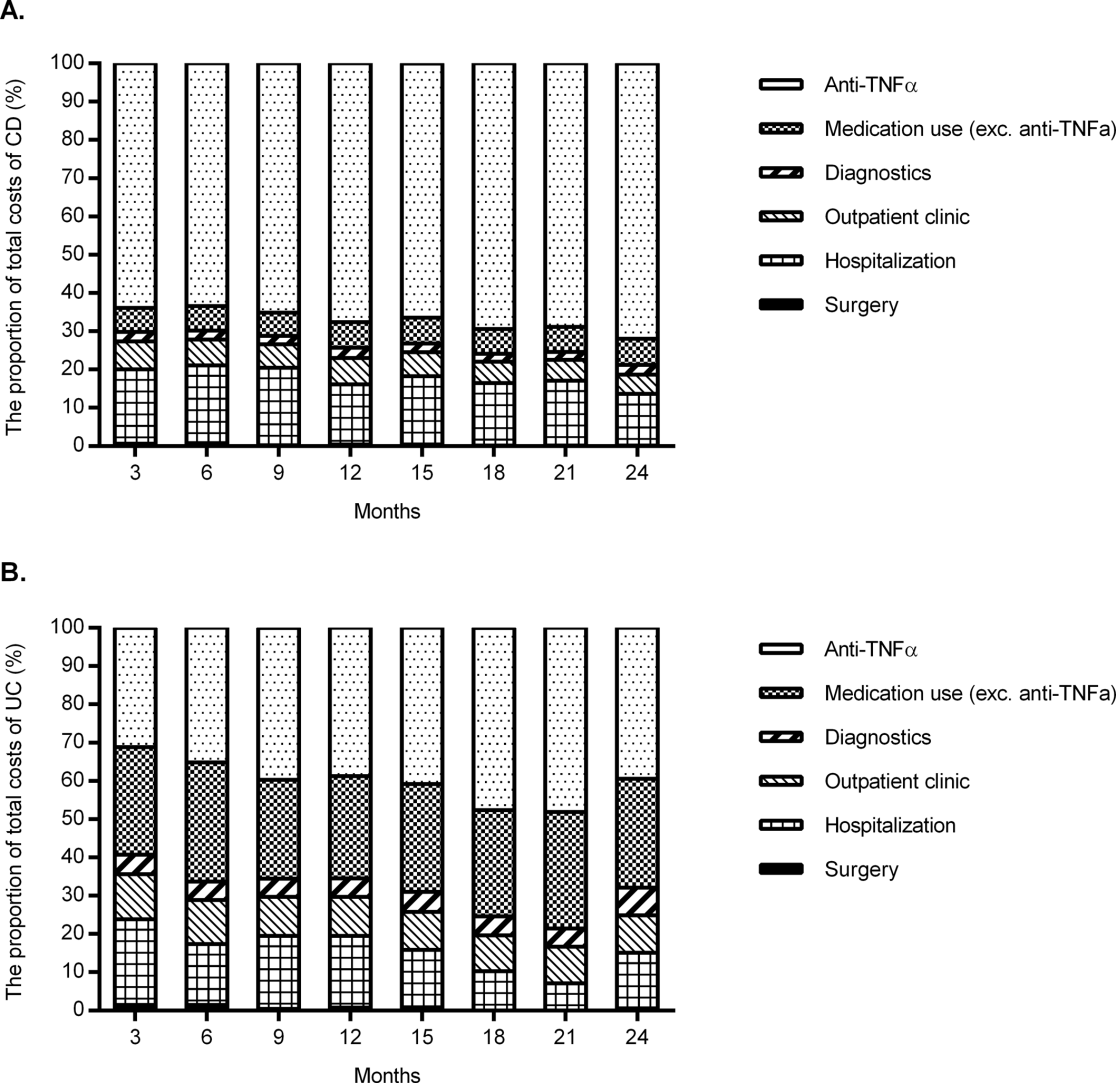
**A**. The proportion of healthcare costs for an average CD-patient over two-year follow up. **B.** The proportion of healthcare costs for an average UC-patient over two-year follow up.

In Fig 3A and 3B, the breakdown of healthcare costs over time in the CD and UC cohorts is depicted. Although the absolute healthcare costs did not change significantly over the two years of follow-up, the proportion of anti-TNF therapy-related costs increased from 64% to 72% in CD (p<0.01), and from 31% to 39% in UC (p<0.01). This was mainly due to an increased use of anti-TNF over two years of follow up. This increase was accompanied by a reduction of the proportion of hospitalization costs, which decreased from 19% to 13% in CD (p<0.01), and from 22% to 15% in UC (p<0.01). The proportion of healthcare costs due to surgery, outpatient clinic, other mediation use and diagnostic procedures remained stable over time (S5C and S5D Table).

### Predictors of healthcare costs

In Table 2 the results of the multivariate analysis on predictors of healthcare costs are shown. In CD, penetrating disease course was associated with an increase of healthcare costs (adjusted odds ratio (Adj. OR) 1.95 (95% CI 1.02–3.37)). Furthermore, anti-TNF therapy (Adj. OR 0.09 (95% CI 0.02–0.12)) and disease activity (0.47 (95% CI 0.24–0.93)) at three months of follow-up were found to be associated with a decrease of healthcare costs over two years of follow-up. This was mainly due to discontinuation of anti-TNF therapy in 20% of CD patients with disease activity. In case of UC, only age <40 years (n = 225, 39.8% of the UC population) was found to independently predict an increase of healthcare costs (adj. OR 4.72 (95% CI 1.61–13.86)). The percentage UC patients <40 years receiving Anti-TNF therapy increased from 4.9% at baseline to 9.9% over two years of follow up.

**Table 2:** Multivariate logistic regression analyses of CD and UC patients with increase of healthcare costs as dependent variable.

	CD		UC	
Variable	Adj. OR (95% CI)	p-value	Adj. OR (95% CI)	p-value
**Age (at 3 months follow up)**				
< 40 years	1.03 (0.54–1.98)	0.93	4.72 (1.61–13.86)	<0.01
>40 years (ref)	1		1	
**Disease duration (at 3 months follow up)**				
< 3 years	0.54 (0.17–1.68)	0.29	2.03 (0.55–7.54)	0.29
>3 years (ref)	1		1	
**Abdominal surgery in the past**				
Yes	0.68 (0.35–1.35)	0.27	3.36 (0.13–1.070)	0.07
No (ref)	1		1	
**Anti-TNF therapy (at 3 months follow up)**				
Yes	0.09 (0.02–0.12)	<0.01	0.14 (0.02–1.40)	0.10
No (ref)	1		1	
**Disease activity (at 3 months follow up)**				
Yes	0.47 (0.24–0.93)	0.03	-	
No (ref)	1			
**Penetrating disease course**				
Yes	1.95 (1.02–3.73)	0.04		
No (ref)	1			

## Discussion

The widespread use of anti-TNF in the treatment of patients with IBD has changed the healthcare landscape radically and has led to a major shift in cost profiles.[1] For the first time, we prospectively show in a large longitudinal study that IBD-related costs remain stable over a period of two years. In this period, we observed an ongoing shift of cost profiles with an increasing proportion of anti-TNF-related healthcare costs and a reduction of hospitalization costs.

Most of the IBD-related costs were incurred by anti-TNF therapy, both in CD and UC patients. The present data underscore our previous observations that healthcare expenditures in IBD shift from costs related to hospitalization and surgery to costs driven by medication use.[1] Due to the differences in study design and study populations, it is difficult to compare our results with other studies. For example, the recently published EPICOM cost data from a population-based inception cohort of patients in the first year after the diagnosis reported that the main cost drivers were investigative procedures (21%), surgical procedures (26%) and anti-TNF therapy (15%).[13] Interestingly, 20% and 4% of their CD and UC patients were already on anti-TNF therapy in the first year after diagnosis, which is almost identical to the rates observed in our cohort (21% in CD and 3% in UC).

An important observation is the ongoing rise of anti-TNF therapy-related costs, with a concurrent reduction of hospitalization costs. A similar trend in increase of anti-TNF therapy-related costs has been found in rheumatoid arthritis.[14;15] In two national registry cost-of-illness studies covering 20-years of follow-up, a downward trend for all costs, apart from the costs for anti-TNF, therapy has been reported. The decline of costs related to hospitalization in IBD is consistent with the observed decrease in surgery and hospitalisation rates in population-based studies.[16;17]

Even though healthcare cost differ to a large extend between Western countries, comparable trends in treatment paradigms should have induced the same alterations in cost profiles as observed in our study. For example, Kappelman et al. studied healthcare costs using medical and pharmacy claims from an administrative database between 2003 and 2004, in which 10% of all CD patients had at least two claims of infliximab infusions.[18] In this study, pharmaceutical claims accounted for the largest proportion of healthcare costs (35%), from which infliximab was the most costly medication.

The large sample size and longitudinal data enabled us to study predictors of healthcare costs over time. In CD, penetrating disease was found to be associated with an increase of costs over two years of follow-up. This can be attributed to the fact that a penetrating disease is a predictor of poor outcome in CD, resulting in frequent surgery and hospitalizations[19–21]. Furthermore, this complication of CD is often treated with anti-TNF compounds (26.9% in our cohort, data not shown).

In UC patients younger than 40 years of age, an increase of healthcare costs was encountered as well. We found a 100% increase of anti-TNF use among young UC patients during two years of follow-up. This finding is in line with previous studies in which younger age in UC was found to be associated with a more severe disease course and an increased risk of relapses.[22–24] Furthermore, young age is associated with more extended colitis in which escalating therapy towards anti-TNF medication or surgery is frequently required.[25] In contrast, anti-TNF therapy and disease activity were associated with a decrease of healthcare costs. This was mainly due to the fact that in these patients, anti-TNF therapy was eventually discontinued. Whether this was due to treatment failure, side effects or cessation of this drug because of treatment success could not be discerned from our data.

Our study has several limitations. First, an inherent limitation of a longitudinal study using a web-based questionnaire design is the high rate of loss to follow-up. We tried to reduce the impact of this problem by using mixed models to correct for missing values. Furthermore, we performed a non-responder study, which showed that responders (i.e. the individuals completing all questionnaires) were older and had a longer disease duration. Since costs in elderly IBD patients are lower than in younger patients,[26] we may have underestimated total healthcare costs. Interestingly, even in this relatively old population, the prescription of anti-TNF therapy increased over a follow-up period of two years. Furthermore, we did not have clinical data such as endoscopic or laboratory markers of disease activity at our disposal. Potentially, these might prove to be important determinants of future healthcare costs as well. For example, deep ulcers or high faecal calprotectin levels may predict a severe disease course with associated high costs.

In conclusion, there is an apparent shift in cost profiles from surgery and hospitalization towards anti-TNF therapy. However, total IBD costs remain remarkably stable over time, suggesting that the anti-TNF-related costs are compensated by a reduction of hospitalization costs. This may corroborate the notion that investment in expensive medical therapy might be cost-effective from a pharmaco-economical point of view,presuming that a reduction in hospital admission is equal with an improvement in quality-of-life. Whether long-term anti-TNF therapy is truly cost-effective in IBD has yet to be determined. Further careful monitoring of changes in the costs of care for IBD patients will aid timely, sensible economic decision-making.

## Supporting Information

S1 Table Unit costs of resource use in Euros for the year 2011. (DOCX)

S2 Table Possible predictive factors for future high costs. (DOCX)

S3 Table Number of responders per time point. (DOCX)

S4 Table Comparison between patients who completed the two year follow up (responders) and patients who were lost to follow up (non-responders). (DOCX)

S5 Table **A.** Average healthcare costs/patients per 3 months in CD patients (€). **B.** Average healthcare costs/patients per 3 months in UC patients (€). **C.** Proportion of healthcare costs in CD (%). **D.** Proportion of healthcare costs in UC (%).
(DOCX)

## Abbreviations

CD: Crohn’s disease
UC: Ulcerative colitis
IBD: Inflammatory bowel disease
Anti-TNF: Anti-tumour necrosis factor α
COIN: Costs Of Inflammatory bowel disease in the Netherlands
DTCs: Diagnosis Treatment Combinations
CT: Computer Tomography
MRI: Magnetic Resonance Imaging
DXA: Dual-emission X-ray absorptiometry
IQR: Interquartile range
SD: Standard deviation
CI: Confidence interval
Adj. OR: Adjusted Odds Ratio
