# Supplementary material for: Evolution of Costs of Inflammatory Bowel Disease over Two Years of Follow-Up
Source: PLoS One. 2016 Apr 21;11(4):e0142481. doi: 10.1371/journal.pone.0142481 (PMC4839678; doi:10.1371/journal.pone.0142481)
Supplement: S1 Table — (DOCX) [file pone.0142481.s001.docx]

**Table S1**. unit costs of resource use for the year 2011

|  | | | **Unit cost price (€)** | |
| --- | --- | --- | --- | --- |
|  | | | Unit cost price per visit | |
| **Outpatient clinic consultations** | | |  | |
|  |  | District hospital | 64.64 | |
|  |  | University medical centre | 130.29 | |
|  | Emergency room | | 152.51 | |
|  | General practitioner | |  | |
|  |  | Visit (day-time) | 28.28 | |
|  |  | Home visit (day-time) | 43.43 | |
|  |  | Visit (weekend/ night-time) | 82.00^a^ | |
|  |  | Home visit (weekend/ night-time) | 123.00^a^ | |
|  |  | | Unit cost price per hour | |
|  | IBD or stoma nurse – per hour | | 44.50 | |
|  | Dietician – per hour | | 48.70 | |
| **Hospitalisation** | | | Unit cost price per day | |
|  | Medical ward | |  | |
|  |  | General hospital | 439.35 | |
|  |  | University medical centre | 580.75 | |
|  | Intensive care unit | | 2204.83 | |
| **Medication use** | | | Unit costs price per 3 months | |
|  | Mesalazine | | UC: 212.42^b^  CD: 246,90^c^ | |
|  | Prednisone | | 15.09 ^d^ | |
|  | Budesonide | | 189.81^e^ | |
|  | Azathioprine - 150 g/day | | 90.62^f^ | |
|  | Mercaptopurine - 50 mg/day | | 90.62^g^ | |
|  | Methotrexate - 15 mg/ week | | 248,44^h^ | |
|  | Infliximab | | 4,853^i^ | |
|  | Adalimumab | | 4,364^j^ | |
| **Surgery** | | | Unit cost price/ surgery ^k^ | |
|  | Ileocecal resection/  resection neoterminal ileum | | 1,184.00 | |
|  | Partial colectomy | | 1,726.00 | |
|  | Subtotal colectomy | | 1,726.00 | |
|  | Abcess surgery | | 168.00 | |
|  | Complex fistula surgery | | 2,302.00 | |
|  | Rectum amputation | | 3,149.00 | |
|  | Ileostomy | | 743.00 | |
| **Diagnostic procedures** | | | Unit cost price/ diagnostic procedure | |
|  | Colonoscopy | | 343.79 | |
|  | CT scan | | 152.96 | |
|  | MRI scan | | 187.96 | |
|  | Abdominal X-ray | | 43.38 | |
|  | Ultrasonography | | 37.67 | |
|  | DXA scan | | 84.47 | |
|  | Laboratory | | 18.06^l^ | |
| **Sick leave from paid work (patient)** | | | Productivity losses per working hour | |
|  |  |  | Females | Males |
|  | 15-19 years | | 8.94 | 9.84 |
|  | 20-24 years | | 17.52 | 18.11 |
|  | 25-29 years | | 24.09 | 24.67 |
|  | 30-34 years | | 28.09 | 30.24 |
|  | 35-39 years | | 29.84 | 34.71 |
|  | 40-44 years | | 29.64 | 37.40 |
|  | 45-49 years | | 29.49 | 39.09 |
|  | 50-54 years | | 29.84 | 39.84 |
|  | 55-59 years | | 30.09 | 40.17 |
|  | 60-64 years | | 29.24 | 39.91 |
| **Sick leave from unpaid work (patient and caregiver)** | | | Productivity losses per working hour | |
|  |  | | 12.96 | |

^a^ Price based on average cost price of 55 general practitioners (weekend/evening/night).

^b^ Price based on average dose of 2000 mg/day during 91 days.

^c^ Price based on average dose of 2400 mg/day during 91 days.

^d^ Price based on average dose of 10 mg/day during 91 days.

^e^ Price based on average dose of 6 g/day during 91 days.

^f^ Price based on average dose of 150 mg/day during 91 days.

^g^ Price based on average dose of 50 mg/day during 91 days.

^h^ Price based on average dose of 15 mg/ week during 13 weeks.

^i^ Price based on average weight of 75 kg and 1.8 infusions per 3 months.

^j^ Price based on 6,5 injections per 3 months (81% administered adalimumab **40 mgs per 2 weeks) or 13 injections per 3 months (19% of patients administered adalimumab 80 mgs per 2 weeks).**

^k^ Days admitted at the surgical or medical were not included in the cost price of surgery, but assessed separately ^.^

^l^ Price based on full blood count and differential, C-reactive protein, alanine aminotransferase, aspartate aminotransferase, y-glutamyl transferase, sodium, potassium, creatinine, albumin.

^m^ For patients with an ileostomy costs for caring for the stoma were based on a standard care package. This is based on the assumption of an exchange of base disk 4 times per week and of the ileostomy bag twice/day.

**Table S2.** Possible predictors for future high costs

|  | **Study references** | | |
| --- | --- | --- | --- |
| **Variable** | **Predictors of healthcare costs** | **Predictors of productivity losses or costs** | **Predictors of poor prognosis** |
| Female gender  Age | (1)  (2;3) | (4)  (2;4;5) | (6;7)  (6-10) |
| Smoking |  |  | (7) |
| Education level |  | (4) |  |
| Short disease duration | (1) |  |  |
| Penetrating disease course | (2;11) |  | (8;10;12) |
| Disease localisation  Disease activity/ flare | (1;2;11) | (2;4;5;11) | (6;9;10;12)  (7;13) |
| Hospitalization | (1) |  |  |
| Surgery |  | (4;5) |  |
| Ileostomy  Anti TNFa therapy | (11)  (1) | (4) |  |
| Steroids |  | (4;5) | (8;12) |
| Joint complaints  Chronic back pain  Depression |  | (4)  (4)  (4) |  |

1. Prenzler A, Bokemeyer B, von der Schulenburg JM, et al. Health care costs and their predictors of inflammatory bowel diseases in Germany. Eur J Health Econ. 2011;12:273-283.

2. Mesterton J, Jonsson L, Almer SH, et al. Resource use and societal costs for Crohn's disease in Sweden. Inflamm Bowel Dis. 2009;15:1882-1890.

3. Kappelman MD, Rifas-Shiman SL, Porter CQ, et al. Direct health care costs of Crohn's disease and ulcerative colitis in US children and adults. Gastroenterology. 2008;135:1907-1913.

4. van der Valk ME, Mangen MJ, Leenders M, et al. Risk factors of work disability in patients with inflammatory bowel disease - A Dutch nationwide web-based survey: Work disability in inflammatory bowel disease. J Crohns Colitis. 2013.

5. Hoivik ML, Moum B, Solberg IC, et al. Work disability in inflammatory bowel disease patients 10 years after disease onset: results from the IBSEN Study. Gut.

6. Henriksen M, Jahnsen J, Lygren I, et al. Ulcerative colitis and clinical course: results of a 5-year population-based follow-up study (the IBSEN study). Inflamm Bowel Dis. 2006;12:543-550.

7. Hoie O, Schouten LJ, Wolters FL, et al. Ulcerative colitis: no rise in mortality in a European-wide population based cohort 10 years after diagnosis. Gut. 2007;56:497-503.

8. Beaugerie L, Seksik P, Nion-Larmurier I, et al. Predictors of Crohn's disease. Gastroenterology. 2006;130:650-656.

9. Wolters FL, Russel MG, Sijbrandij J, et al. Phenotype at diagnosis predicts recurrence rates in Crohn's disease. Gut. 2006;55:1124-1130.

10. Solberg IC, Vatn MH, Hoie O, et al. Clinical course in Crohn's disease: results of a Norwegian population-based ten-year follow-up study. Clin Gastroenterol Hepatol. 2007;5:1430-1438.

11. van der Valk ME, Mangen MJ, Leenders M, et al. Healthcare costs of inflammatory bowel disease have shifted from hospitalisation and surgery towards anti-TNFalpha therapy: results from the COIN study. Gut. 2012.

12. Loly C, Belaiche J, Louis E. Predictors of severe Crohn's disease. Scand J Gastroenterol. 2008;43:948-954.

13. Solberg IC, Lygren I, Jahnsen J, et al. Clinical course during the first 10 years of ulcerative colitis: results from a population-based inception cohort (IBSEN Study). Scand J Gastroenterol. 2009;44:431-440.

**Table S3.** Number of responders per time point

| **Time point** | **Number of CD patients** | **Number of**  **UC patients** |
| --- | --- | --- |
| **Baseline** | 1,558 | 1,056 |
| **3 months** | 1,307 | 915 |
| **6 months** | 918 | 640 |
| **9 months** | 859 | 640 |
| **12 months** | 938 | 700 |
| **15 months** | 917 | 682 |
| **18 months** | 879 | 643 |
| **21 months** | 842 | 619 |
| **24 months** | 736* | 566* |

Response rate CD: 47%, UC 54%

**Table S4.** Comparison between patients who completed the two year follow up (responders) and patients who were lost to follow up (non responders)

|  | **CD** | | **UC** | |
| --- | --- | --- | --- | --- |
|  | **Responders**  **n=737** | **Non-responders**  **n=821** | **Responders**  **n= 566** | **Non-responders**  **n=490** |
| **Male gender (%)** | 295 (40.0) | 279 (34.0) | 300 (53.0) | 228 (46.5) |
| **Age – years (± SD)** | 50.5 (13.5) | 45.6 (13.8) | 52.4 (12.9) | 48.0 (13.7) |
| **Disease duration – median (IQR)** | 18.2 (10.1-18.2) | 16.8 (11.4) | 16.0 (9.0-16.0) | 13.9 (10.0) |
| **Disease localisation (%)** |  |  |  |  |
| Large bowel | 204 (27.7) | 227 (27.6) | 566 (100) | 490 (100) |
| Small bowel | 152 (20.6) | 154 (18.8) | n/a | n/a |
| Both small and large bowel | 361 (49.0) | 407 (49.6) | n/a | n/a |
| Unknown | 20 (2.7) | 33 (4.0) | n/a | n/a |
| **Penetrating disease course (%)** | 348 (47.2) | 396 (48.2) | n/a | n/a |
| **Disease activity (%)** | 618 (16.1) | 117 (14.3) | 452 (20.1) | 98 (20.0) |
| **Abdominal surgery (%)** | 416 (56.4) | 427 (52.0) | 106 (21.3) | 89 (18.2) |

SD: Standard deviation; IQR: interquartile range; n/a: not applicable

**Table S5A**. Average healthcare costs/patient per 3 months in CD patients (€)

| **Months** | **Surgery** | **Hospitalization** | **Outpatient clinic** | **Diagnostic procedures** | **Medication use (exc. Anti-TNFa)** | **Anti-TNFa** |
| --- | --- | --- | --- | --- | --- | --- |
| **3** | 996 | 319.23 | 119.93 | 40.81 | 103.84 | 1,048.1 |
| **6** | 13.33 | 334.31 | 111.47 | 38.14 | 106.12 | 1,042.55 |
| **9** | 3.34 | 340.89 | 102.75 | 35.9 | 103.09 | 1,093.27 |
| **12** | 7.63 | 244 | 107.52 | 41.36 | 103.97 | 1,053.93 |
| **15** | 7.41 | 275.51 | 96.78 | 35.06 | 103.62 | 1,027.32 |
| **18** | 1.53 | 251.89 | 86.66 | 32.65 | 100.53 | 1,070.62 |
| **21** | 2.24 | 268.91 | 85.61 | 33.66 | 102.28 | 1,090.59 |
| **24** | 1.83 | 195.61 | 74.1 | 36.34 | 99.24 | 1,044.54 |

**Table S5B.** Average healthcare costs/patients per 3 months in UC patients (€)

| **Months** | **Surgery** | **Hospitalization** | **Outpatient clinic** | **Diagnostic procedures** | **Medication use (exc. Anti-TNFa)** | **Anti-TNFa** |
| --- | --- | --- | --- | --- | --- | --- |
| **3** | 8,36 | 130,58 | 68,46 | 29,95 | 163,87 | 181.35 |
| **6** | 8,09 | 87,89 | 63,67 | 26,81 | 172,27 | 194.09 |
| **9** | 2,7 | 122,25 | 64,56 | 30,7 | 164,98 | 253.22 |
| **12** | 5,17 | 118,17 | 63,97 | 30,37 | 169,29 | 243.99 |
| **15** | 5,06 | 87,24 | 58,05 | 30,68 | 163,78 | 237.63 |
| **18** | 0 | 60,47 | 55,21 | 29,33 | 163,73 | 280.71 |
| **21** | 0 | 37,19 | 50,37 | 24,51 | 159,47 | 251.6 |
| **24** | 3,05 | 82,87 | 55,23 | 40,84 | 161,56 | 223.72 |

**Table S5C**. Proportion of healthcare costs in CD (%)

| **Months** | **Surgery** | **Hospitalization** | **Outpatient clinic** | **Diagnostic procedures** | **Medication use (exc. Anti-TNFa)** | **Anti-TNFa** |
| --- | --- | --- | --- | --- | --- | --- |
| **3** | 0.61 | 19.44 | 7.30 | 2.49 | 6.32 | 63.84 |
| **6** | 0.81 | 20.31 | 6.77 | 2.32 | 6.45 | 63.34 |
| **9** | 0.20 | 20.30 | 6.12 | 2.14 | 6.14 | 65.11 |
| **12** | 0.49 | 15.66 | 6.90 | 2.65 | 6.67 | 67.63 |
| **15** | 0.48 | 17.82 | 6.26 | 2.27 | 6.70 | 66.46 |
| **18** | 0.10 | 16.32 | 5.61 | 2.11 | 6.51 | 69.35 |
| **21** | 0.14 | 16.98 | 5.41 | 2.13 | 6.46 | 68.88 |
| **24** | 0.13 | 13.47 | 5.10 | 2.50 | 6.84 | 71.95 |

**Table S5D**. Proportion of healthcare costs in UC (%)

| **Monthts** | **Surgery** | **Hospitalization** | **Outpatient clinic** | **Diagnostic procedures** | **Medication use (exc. Anti-TNFa)** | **Anti-TNFa** |
| --- | --- | --- | --- | --- | --- | --- |
| **3** | 1.44 | 22.41 | 11.75 | 5.14 | 28.13 | 31.13 |
| **6** | 1.46 | 15.90 | 11.52 | 4.85 | 31.16 | 35.11 |
| **9** | 0.42 | 19.15 | 10.11 | 4.81 | 25.84 | 39.66 |
| **12** | 0.82 | 18.73 | 10.14 | 4.81 | 26.83 | 38.67 |
| **15** | 0.87 | 14.98 | 9.97 | 5.27 | 28.12 | 40.80 |
| **18** | 0.00 | 10.26 | 9.37 | 4.98 | 27.78 | 47.62 |
| **21** | 0.00 | 7.11 | 9.63 | 4.69 | 30.48 | 48.09 |
| **24** | 0.54 | 14.61 | 9.74 | 7.20 | 28.48 | 39.44 |
