# Supplementary material for: Evolution of Costs of Inflammatory Bowel Disease over Two Years of Follow-Up
Source: PLoS One. 2016 Apr 21;11(4):e0142481. doi: 10.1371/journal.pone.0142481 (PMC4839678; doi:10.1371/journal.pone.0142481)
Supplement: S3 Table — (DOCX) [file pone.0142481.s003.docx]

**Table S3.** Number of responders per time point

| **Time point** | **Number of CD patients** | **Number of**  **UC patients** |
| --- | --- | --- |
| **Baseline** | 1,558 | 1,056 |
| **3 months** | 1,307 | 915 |
| **6 months** | 918 | 640 |
| **9 months** | 859 | 640 |
| **12 months** | 938 | 700 |
| **15 months** | 917 | 682 |
| **18 months** | 879 | 643 |
| **21 months** | 842 | 619 |
| **24 months** | 736* | 566* |

Response rate CD: 47%, UC 54%
