# Supplementary material for: Evolution of Costs of Inflammatory Bowel Disease over Two Years of Follow-Up
Source: PLoS One. 2016 Apr 21;11(4):e0142481. doi: 10.1371/journal.pone.0142481 (PMC4839678; doi:10.1371/journal.pone.0142481)
Supplement: S4 Table — (DOCX) [file pone.0142481.s004.docx]

**Table S4.** Comparison between patients who completed the two year follow up (responders) and patients who were lost to follow up (non responders)

|  | **CD** | | **UC** | |
| --- | --- | --- | --- | --- |
|  | **Responders**  **n=737** | **Non-responders**  **n=821** | **Responders**  **n= 566** | **Non-responders**  **n=490** |
| **Male gender (%)** | 295 (40.0) | 279 (34.0) | 300 (53.0) | 228 (46.5) |
| **Age – years (± SD)** | 50.5 (13.5) | 45.6 (13.8) | 52.4 (12.9) | 48.0 (13.7) |
| **Disease duration – median (IQR)** | 18.2 (10.1-18.2) | 16.8 (11.4) | 16.0 (9.0-16.0) | 13.9 (10.0) |
| **Disease localisation (%)** |  |  |  |  |
| Large bowel | 204 (27.7) | 227 (27.6) | 566 (100) | 490 (100) |
| Small bowel | 152 (20.6) | 154 (18.8) | n/a | n/a |
| Both small and large bowel | 361 (49.0) | 407 (49.6) | n/a | n/a |
| Unknown | 20 (2.7) | 33 (4.0) | n/a | n/a |
| **Penetrating disease course (%)** | 348 (47.2) | 396 (48.2) | n/a | n/a |
| **Disease activity (%)** | 618 (16.1) | 117 (14.3) | 452 (20.1) | 98 (20.0) |
| **Abdominal surgery (%)** | 416 (56.4) | 427 (52.0) | 106 (21.3) | 89 (18.2) |

SD: Standard deviation; IQR: interquartile range; n/a: not applicable
