# Supplementary material for: Evolution of Costs of Inflammatory Bowel Disease over Two Years of Follow-Up
Source: PLoS One. 2016 Apr 21;11(4):e0142481. doi: 10.1371/journal.pone.0142481 (PMC4839678; doi:10.1371/journal.pone.0142481)
Supplement: S5 Table — A. Average healthcare costs/patients per 3 months in CD patients (€). B. Average healthcare costs/patients per 3 months in UC patients (€). C. Proportion of healthcare costs in CD (%). D. Proportion of healthcare costs in UC (%). (DOCX) [file pone.0142481.s005.docx]

**Table S5A**. Average healthcare costs/patient per 3 months in CD patients (€)

| **Months** | **Surgery** | **Hospitalization** | **Outpatient clinic** | **Diagnostic procedures** | **Medication use (exc. Anti-TNFa)** | **Anti-TNFa** |
| --- | --- | --- | --- | --- | --- | --- |
| **3** | 996 | 319.23 | 119.93 | 40.81 | 103.84 | 1,048.1 |
| **6** | 13.33 | 334.31 | 111.47 | 38.14 | 106.12 | 1,042.55 |
| **9** | 3.34 | 340.89 | 102.75 | 35.9 | 103.09 | 1,093.27 |
| **12** | 7.63 | 244 | 107.52 | 41.36 | 103.97 | 1,053.93 |
| **15** | 7.41 | 275.51 | 96.78 | 35.06 | 103.62 | 1,027.32 |
| **18** | 1.53 | 251.89 | 86.66 | 32.65 | 100.53 | 1,070.62 |
| **21** | 2.24 | 268.91 | 85.61 | 33.66 | 102.28 | 1,090.59 |
| **24** | 1.83 | 195.61 | 74.1 | 36.34 | 99.24 | 1,044.54 |

**Table S5B.** Average healthcare costs/patients per 3 months in UC patients (€)

| **Months** | **Surgery** | **Hospitalization** | **Outpatient clinic** | **Diagnostic procedures** | **Medication use (exc. Anti-TNFa)** | **Anti-TNFa** |
| --- | --- | --- | --- | --- | --- | --- |
| **3** | 8,36 | 130,58 | 68,46 | 29,95 | 163,87 | 181.35 |
| **6** | 8,09 | 87,89 | 63,67 | 26,81 | 172,27 | 194.09 |
| **9** | 2,7 | 122,25 | 64,56 | 30,7 | 164,98 | 253.22 |
| **12** | 5,17 | 118,17 | 63,97 | 30,37 | 169,29 | 243.99 |
| **15** | 5,06 | 87,24 | 58,05 | 30,68 | 163,78 | 237.63 |
| **18** | 0 | 60,47 | 55,21 | 29,33 | 163,73 | 280.71 |
| **21** | 0 | 37,19 | 50,37 | 24,51 | 159,47 | 251.6 |
| **24** | 3,05 | 82,87 | 55,23 | 40,84 | 161,56 | 223.72 |

**Table S5C**. Proportion of healthcare costs in CD (%)

| **Months** | **Surgery** | **Hospitalization** | **Outpatient clinic** | **Diagnostic procedures** | **Medication use (exc. Anti-TNFa)** | **Anti-TNFa** |
| --- | --- | --- | --- | --- | --- | --- |
| **3** | 0.61 | 19.44 | 7.30 | 2.49 | 6.32 | 63.84 |
| **6** | 0.81 | 20.31 | 6.77 | 2.32 | 6.45 | 63.34 |
| **9** | 0.20 | 20.30 | 6.12 | 2.14 | 6.14 | 65.11 |
| **12** | 0.49 | 15.66 | 6.90 | 2.65 | 6.67 | 67.63 |
| **15** | 0.48 | 17.82 | 6.26 | 2.27 | 6.70 | 66.46 |
| **18** | 0.10 | 16.32 | 5.61 | 2.11 | 6.51 | 69.35 |
| **21** | 0.14 | 16.98 | 5.41 | 2.13 | 6.46 | 68.88 |
| **24** | 0.13 | 13.47 | 5.10 | 2.50 | 6.84 | 71.95 |

**Table S5D**. Proportion of healthcare costs in UC (%)

| **Monthts** | **Surgery** | **Hospitalization** | **Outpatient clinic** | **Diagnostic procedures** | **Medication use (exc. Anti-TNFa)** | **Anti-TNFa** |
| --- | --- | --- | --- | --- | --- | --- |
| **3** | 1.44 | 22.41 | 11.75 | 5.14 | 28.13 | 31.13 |
| **6** | 1.46 | 15.90 | 11.52 | 4.85 | 31.16 | 35.11 |
| **9** | 0.42 | 19.15 | 10.11 | 4.81 | 25.84 | 39.66 |
| **12** | 0.82 | 18.73 | 10.14 | 4.81 | 26.83 | 38.67 |
| **15** | 0.87 | 14.98 | 9.97 | 5.27 | 28.12 | 40.80 |
| **18** | 0.00 | 10.26 | 9.37 | 4.98 | 27.78 | 47.62 |
| **21** | 0.00 | 7.11 | 9.63 | 4.69 | 30.48 | 48.09 |
| **24** | 0.54 | 14.61 | 9.74 | 7.20 | 28.48 | 39.44 |
